# Readiness of University Students in Riyadh to Participate in Basic Life Support Programs: A Cross-Sectional Study

**DOI:** 10.7759/cureus.64749

**Published:** 2024-07-17

**Authors:** Lama A Alzelfawi, Norah I Alhumaidan, Lena M AlDosari, Ghadah F Aldayel, Nora M Alzoum, Rahaf B Alsliham, Afnan A Alawadh, Dimah M AlMazyad, Amjad A Alrizqi, Doaa S Abdelrahman, Amel A Fayed, Amal M Goda

**Affiliations:** 1 Medicine, Princess Nourah Bint Abdulrahman University, Riyadh, SAU; 2 Clinical Sciences, Princess Nourah Bint Abdulrahman University, Riyadh, SAU; 3 Public Health, Faculty of Medicine, Ain Shams University, Riyadh, SAU

**Keywords:** saudi arabia, awareness, readiness, willingness, basic life support

## Abstract

Introduction

Out-of-hospital cardiac arrest is one of the greatest causes of death in the world. When basic life support (BLS) techniques are performed rapidly, the odds of survival increase. The aim of this research is to assess the university students' preparation and knowledge level regarding their interest in participating in BLS.

Methods

A cross-sectional study using an online self-administered questionnaire was conducted between January 20 and March 20, 2022. The questionnaire addressed awareness about BLS, willingness to participate in such courses, perceived barriers and incentives, and course uptake, in addition to the sociodemographic profile of participants. Logistic regression analysis was used to identify the factors significantly associated with the willingness to participate in BLS courses and the associations are reported as adjusted odds ratios (AORs) with 95% confidence intervals (CIs).

Results

A total of 1,546 students completed the questionnaire; almost half of them (n=761, 49.2%) were aged 17 to 21 and the majority were females (n=1,132, 73.2%). Only one-third of the population had heard about BLS (n=519, 33.6%), 27.1% (n=419) recognized where to register for BLS training, and 20.3% (n=314) had taken a BLS course. Most participants (n=1,081, 69.9%) demonstrated a low level of awareness. Conversely, a substantial portion (n=1,204, 77.9%) expressed a personal interest in undertaking the course. It was observed that participants who are affiliated with healthcare specialties (AOR = 5.96, 95% CI = 4.66-7.63, p<0.05) exhibited greater knowledge about BLS, while females (OR = 2.49, 95% CI = 1.52-4.08, p<0.05) and those in healthcare specialties (OR = 2.23, 95% CI = 1.29-3.82, p<0.05) displayed a notably higher inclination to enroll in BLS courses compared to their counterparts.

Conclusion

Despite the limited awareness of BLS among university students, there is a strong willingness to engage in BLS courses. It is crucial to motivate students to partake in these courses and emphasize the availability of accredited centers for their education institutes.

## Introduction

Cardiac arrest, both in and out of hospital settings, constitutes a critical and potentially fatal medical emergency, contributing to 15% of global mortality rates. Individuals with pre-existing cardiovascular conditions face a higher risk of experiencing cardiac arrest [1,2]. Out-of-hospital cardiac arrest (OHCA) presents a significant worldwide challenge, with an average incidence of 55 cases per 100,000 person-years in adults. In the USA and Europe, OHCA leads to 276,000 and 350,000 annual fatalities, respectively. In Saudi Arabia, survival rates for cardiac arrests are notably low [3,4]. The Swift initiation of basic life support (BLS) through the prompt activation of the chain of survival is paramount in improving survival rates, potentially up to 70% in these cases [5,6]. Each minute of delay after sudden cardiac arrest onset diminishes resuscitation benefits by about 10%; moreover, the rapid initiation of the chain of survival involves recognizing the event early, activating the emergency response system, commencing cardiopulmonary resuscitation, administering defibrillation using automated external defibrillators (AEDs), and securing advanced medical care. All steps must be executed accurately for optimal outcomes [7,8]. Additionally, it is crucial for the entire community, especially healthcare professionals, to be well-versed in both BLS and advanced cardiac life support [9,10]. Despite numerous studies affirming that imparting BLS training through educational programs to non-medical individuals significantly enhances pre-hospital resuscitation outcomes, there persists a knowledge deficit, particularly among university students who are considered highly prone to encountering emergencies and instances of OHCA [11-13]. Moreover, nationally and internationally, investigations into BLS awareness among healthcare professionals and students have consistently revealed subpar knowledge levels [14-16]. Presently, there exists a paucity of data assessing the readiness of university students in Riyadh regarding BLS courses. Given the city's numerous universities, this study seeks to evaluate the preparedness of university students in Riyadh to engage in BLS educational activities, encompassing an analysis of their knowledge, readiness, and potential barriers to participation in such initiatives.

## Materials and methods

Study design, participants, and setting

Data for the study were collected utilizing the Research Electronic Data Capture (REDCap) electronic data collection tools (Vanderbilt University, TN, USA), which were hosted at Princess Nourah Bint Abdulrahman University (PNU), Riyadh, Saudi Arabia. REDCap is a secure online software platform specifically designed to facilitate data collection for research studies. In this study, we utilized a cross-sectional research design along with a snowball convenience sampling approach. Initially, the research team disseminated the survey link to their primary contacts (university students aged 17 and above from different specialties) through a variety of social networks and the official email service at PNU from January 2022 to March 2022. These initial participants were then encouraged to share the survey with their own networks, and so on. Due to the self-selected and non-probabilistic nature of the sample, it is crucial to highlight that invitations and response rates could not be quantified in accordance with the reporting guidelines of the American Association for Public Opinion Research.

Sample size calculation

Sample size calculation was done using G power application (Heinrich Heine University, Düsseldorf, Germany), the minimum required size for the current study was 1,282 considering the following assumptions: the power of the study is 90% (beta is 0.1) and the level of confidence is 95% (alpha is 0.05), the margin of error was 5%, and the estimated prevalence of excellent BLS awareness among university students was 45%.

Study outcomes and variables

A questionnaire was developed and adapted from previously validated questionnaires from similar studies that assessed BLS knowledge [16,17]. It comprised three main sections: the first section focused on the sociodemographic data, including gender, age, nationality, family income, and specialty, and the second section contained 30 multiple-choice questions assessing the knowledge about BLS steps and techniques along with illustrations, if applicable. The correct answer was given a value of one, while incorrect replies were given a value of zero. As so, less than 60% of the total score (22 out of 30) was considered the cutoff for a poor awareness score.

The third section contained a total of 10 barriers and incentives affecting the willingness to take a BLS course using a 5-point Likert scale ranging from “strongly disagree to strongly agree.” Additional questions were incorporated to explore participants' inclination to enroll in the course, their prior experience with similar courses, as well as instances where they encountered emergencies necessitating BLS. Additionally, inquiries were made about the accessibility of BLS courses in universities or nearby institutions.

Questionnaire validation

To ensure the reliability and validity of the adapted questionnaire, a detailed validation process was undertaken. The initial draft of the questionnaire was reviewed by an expert in the field to ensure content validity, and their feedback was used to refine the questionnaire items. The questionnaire utilized in this study was derived from well-established, previously validated instruments. Although this study did not conduct pilot testing, the adaptation process was carefully managed to maintain the original instruments' integrity and validity.

Statistical analysis

We used SPSS version 21 (Released 2012. IBM SPSS Statistics for Windows, Version 21.0. IBM Corp., Armonk, NY) to analyze the data. Descriptive analysis was used to describe the demographic characteristics. After confirming the normality of variables, a t-test and ANOVA test were used to examine the association between quantitative variables. The chi-square test was used to investigate the relation between categorical variables. Logistic regression analysis was employed to identify the factors significantly associated with the acceptable willingness and intention to get BLS courses. The associations are reported as adjusted odds ratios (AORs) with 95% confidence intervals (CIs) after adjustment for confounders, including sociodemographic and occupational factors. All tests were two-tailed, and p-values less than 0.05 were considered statistically significant.

Ethical consideration

Before starting the study, ethical approval was obtained from the Institutional Review Board of PNU (IRB: 22-0031). Individuals were requested to participate after being given a detailed description of the study objectives. Participants who volunteered to take part in this research project had the right to withdraw at any time with no obligation or affection. The questionnaire did not contain the names or details of the participants to maintain confidentiality.

## Results

A total of 1,546 students completed the questionnaire; almost half of them (n=761, 49.2%) were aged 17 to 21 and most of the participants were females (n=1,132, 73.2%). Students were distributed between different colleges; however, many students were from non-health colleges (n=945, 61.1%). Regarding family income, approximately 55.6% (n=860) of students declared high income (more than 11,000 SR per month nearly 3,000$) (Table 1).

**Table 1:** Demographic characteristics of the studied sample (n=1,546). (N Frequency), (%) Frequency

Variables	N (%)
Age	
17- 21 years old	761 (49.2)
22 and above	785 (50.7)
Gender	
Female	1,132 (73.2)
Male	414 (26.8)
Nationality	
Saudi	1,475 (95.4)
Non-Saudi	71 (4.6)
Family Income	
Less than 5,000 SR	305 (19.7)
5,000-10,000 SR	383 (24.8)
Above 11,000 SR	860 (55.6)
Specialty	
Health-related	601 (38.9)
Non-health related	945 (61.1)

Table 2 shows participants’ experiences with BLS. Nearly one-third of participants (n=519, 33.6%) had heard about BLS, 20.3% (n=314) had taken a BLS course with 34.6% (n=535) of the students had access to the course provided by their university, and 16.6% (n=257) of participants encountered a situation that required BLS. Moreover, 29.3% (n=453) of the knowledge was gained through universities, schools, or online courses, while movies or TV shows were the least sources of knowledge to be reported (n=107, 6.9%). The summative scores from various knowledge items were classified into two groups: those with a poor level of awareness and those with a good level of awareness. In general, a significant majority of participants (n=1,081, 69.9%) exhibited a low level of BLS awareness.

**Table 2:** Participants' experience and awareness with basic life support (BLS). (N Frequency), (%) Frequency

Variables	N (%)
I heard about BLS	519 (33.6)
I took a BLS training course	314 (20.3)
The university provides BLS course	535 (34.6)
I know where to register for a BLS training course in Riyadh	419 (27.1)
I have been in a situation that required me to do BLS	257 (16.6)
Source of knowledge	
Health center or public health campaign	230 (14.9)
University, school, online course	453 (29.3)
Internet (social media, website, etc.)	308 (19.9)
Movie or TV shows	107 (6.9)
Family member, friend, etc.	139 (9.0)
Others	48 (3.1)
Awareness categories	
Poor awareness	1,081 (69.9)
Good awareness	465 (30.1)

Students aged 17-21 years had significantly better awareness scores (M=11.1, SD=3.15) compared to those older than 22 years (M=10.7, SD=3.23), and females had significantly higher awareness scores than males (p=0.02). As for students’ specialties, health college students had significantly higher awareness scores (M=12.57, SD=3.22) as opposed to non-health college students (M=9.85, SD=2.69) (p<0.001). In addition, students with a monthly family income above 11,000 SR had a better awareness score (M=11.1, SD=3.27) significantly (p=0.002). In contrast, no notable differences were found between Saudis and non-Saudis. When examining various knowledge sources, individuals who obtained their knowledge from health centers (M=12.7, SD=3.48) or universities (M=13.01, SD=3.21) demonstrated superior knowledge and awareness regarding BLS in comparison to those who acquired it from other outlets (p<0.001). Regarding course availability within colleges, students who had access to BLS courses through their educational institution (M=12.2, SD=3.50) and those who were aware of where to enroll for such courses (M=12.7, SD=3.6) displayed higher overall scores significantly compared to other groups (p<0.001). As anticipated, participants who had already completed BLS exhibited significantly greater knowledge compared to their counterparts who had not completed the courses (p<0.001).

**Table 3:** Average knowledge scores distribution among various groups of the studied sample. (M Mean), (SD) Standard Deviation, (a) Independent sample t-test, (b) ANOVA Test. *p < 0.05 is significant.

Variable	M (±SD)	P-value
Age		
17-21 years old (n=761)	11.10±3.15	*0.02_a_
22 and above (n=785)	10.73±3.23	
Gender		
Females (n=1,132)	10.79±3.18	*0.02_a_
Males (n=414)	11.24±3.23	
Nationality		
Saudi (n=1,475)	10.91±3.20	0.84_a_
Non-Saudi (n=71)	10.93±3.23	
Specialty		
Health (n=601)	12.57±3.22	*<0.001_a_
Non-health (n=945)	9.85±2.69	
Monthly family income		
Less than 5,000 SR (n=304)	10.54±3.23	*0.002_b_
5,000-10,000 SR (n=383)	10.63±2.95	
Above 11,000 SR (n=859)	11.17±3.27	
Source of knowledge		
Health center or public health campaign (n=231)	12.78±3.48	
University, school, etc. (n=453)	13.01±3.21	*<0.001_b_
Internet (n=308)	11.92±3.25	
Movies or TV shows (n=106)	11.80±3.27	
Family member, friend (n=139)	10.80±3.54	
Availability of BLS course in university		
The course is available (n=535)	12.24±3.50	*<0.001_a_
The course is not available (n=1,011)	10.20±2.78	
I know where to register for a BLS course		
Yes (n=419)	12.78±3.60	*<0.001_a_
No (n=1,127)	10.21±2.73	
I took a BLS course		
Yes (n=314)	13.61±3.23	*<0.001_b_
No, but I want to (n=558)	10.56±2.98	
No, I do not want to (n=83)	10.33±3.21	
I have never heard of it (n=591)	9.89±2.52	

After excluding individuals who had already completed the BLS course (314) and those who were unfamiliar with BLS (591), we found that out of 641 participants (n=558, 87.1%) expressed a desire to enroll in the BLS course, while only 12.9% (n=83) participants stated that they had no intention to do so. Female participants displayed a higher inclination (n=581, 90.1%) compared to their male counterparts (n=515, 79.8%) significantly. Furthermore, there was a notable difference in willingness observed between participants in health-related specialties (n=586, 90.9%) and those in non-health specialties (n=546, 84.7%) (p<0.05). Notably, factors such as age, nationality, family income, and the availability of the course at the university did not exert a significant influence on the decision to pursue a BLS course (Table 4).

**Table 4:** Willingness to take basic life support among various subgroups included in the sample (n=641). The p-value is calculated with Chi-Square and Fisher’s Exact Test. *p<0.05 is significant. This dataset does not include students who already received the BLS course.

Variable	Not Willing	Willing	P-value
Age			
17-21 years old (n=347)	46 (13.3%)	301 (86.7%)	0.81
22 and above (n=294)	37 (12.6%)	257 (87.4%)	
Gender			
Females (n=453)	45 (9.9%)	408 (90.1%)	*<0.01
Males (n=188)	38 (20.2%)	150 (79.8%)	
Nationality			
Saudi (n=605)	77 (12.7%)	528 (87.3%)	0.49
Non-Saudi (n=36)	6 (16.7%)	30 (83.3%)	
Specialty			
Health (n=242)	22 (9.1%)	220 (90.9%)	*0.02
Non-health (n=399)	61 (15.3%)	338 (84.7%)	
Family income			
Less than 5,000 SR (n=132)	17 (12.9%)	115 (87.1%)	0.10
5,000-10,000 SR (n=176)	15 (8.5%)	161 (91.5%)	
Above 11,000 SR (n=333)	51 (15.3%)	282 (84.7%)	
Availability of BLS course in university			
The course is available (n=229)	31 (13.5%)	198 (86.5%)	0.74
The course is not available (n=412)	52 (12.6%)	360 (87.4%)	
I Faced a situation that required BLS			
Yes (n=141)	13 (9.2%)	128 (90.8%)	0.14
No (n=500)	70 (14%)	430 (86%)	

Table 5 shows the barriers and incentives influencing participants' decisions regarding enrolling in a BLS course. Most students exhibited a favorable attitude, with 77.9% (n=1,204) expressing a personal interest in taking the course, and 87.8% (n=1,357) recognizing its potential to aid others. In terms of barriers, 55.3% (n=855) of participants stated that they were unsure about where to access the course, while 50.1% (n=775) believed that it would require a significant time commitment. Additionally, 40% (n=618) felt that the course was not mandatory, and thus, unnecessary. Financial constraints were cited by 38.9% (n=601) of participants as a potential barrier.

**Table 5:** Barriers and incentives of taking basic life support course (n=1,546). (N Frequency), (%) Frequency

Variables	Agree/strongly agree, N (%)
Barriers	
The course takes a lot of time	775 (50.1)
The locations where the course is given are not well known	855 (55.3)
I am too young to take the course	124 (8)
The course is expensive	601 (38.9)
There is no obligation to take the course	618 (40)
The course is difficult for me to take	257 (16.6)
Incentives	
I am personally interested in taking the course	1,204 (77.9)
The course is very related to my specialty	666 (43.1)
The course is mandatory for my career	620 (40.1)
Taking the course will make me able to help others	1,357 (87.8)
I have been in a situation that required basic life support	507 (32.8)

After accounting for various sociodemographic factors through multiple regression models, the level of awareness was independently influenced by the participants' field of specialty. Those in health-related fields demonstrated a significantly higher likelihood of possessing greater knowledge compared to their peers in non-health-related fields (AOR = 5.96, 95% CI = 4.66-7.63) (p<0.05). Furthermore, female participants exhibited a significantly greater willingness to engage in BLS courses (AOR = 2.49, 95% CI = 1.52-4.08) (p<0.05). Additionally, participants in health-related specialties displayed a higher inclination towards participation (AOR = 2.23, 95% CI = 1.29-3.82) (p<0.05) (Table 6).

**Table 6:** Multiple logistic regression for the level of knowledge and willingness to enroll to BLS course. *p<0.05, (CI) confidence interval, (AOR) Adjusted odds ratio, (COR) Crude Odds ratio

Variable		Knowledge			Willingness		
		COR	AOR	CI (95%)	COR	AOR	CI (95%)
Age	17-21 years old	1	1	-	1	1	-
	22-24 years old	0.87	0.96	(0.69-1.1.07)	0.99	1.26	(0.72-2.18)
Gender	Male	1	1	-	1	1	-
	Female	0.78	1.05	(0.80-1.38)	2.30	2.49	(1.52-4.08)*
Nationality	Non-Saudi	1	1	-	1	1	-
	Saudi	0.96	1.23	(0.70-2.17)	1.37	1.85	(0.71-4.83)
Specialty	Non-health	1	1	-	1	1	-
	Health	6.33	5.96	(4.66-7.63)*	1.81	2.23	(1.29-3.82)*
Family income	Less than 5,000 SR	1	1	-	1	1	-
	5,000-10,000SR	0.96	0.98	(0.67-1.42)	1.59	1.52	(0.72-3.22)
	Above 11,000	1.34	1.35	(0.98-1.86)	0.82	0.79	(0.43-1.44)

## Discussion

The current study aimed to assess the awareness and preparedness of university students in Riyadh regarding BLS. The findings revealed a substantial lack of awareness among the participants regarding BLS, with 69.9% (n=1,081) demonstrating low awareness. The poor awareness of BLS knowledge was found in many national and international studies for as in Saudi Arabia, Jordan, Iran, India, Egypt, and the United Kingdom which reflect the widespread of the problem globally [3,14,18-22]. In the current study, the primary knowledge source was official channels such as universities, schools, public courses, and health campaigns. Notably, participants who obtained their knowledge from these official sources demonstrated significantly better awareness of BLS. This underscores the importance of organizing BLS courses on campus with clear and widespread promotion. This finding aligns with a study by Jarrah et al. in Jordan, which identified schools and universities as the main providers of BLS information and training. This emphasizes the necessity for structured BLS courses facilitated through educational institutions, which can be more impactful in enhancing BLS awareness [13]. Furthermore, the current study identified a correlation between increased awareness of BLS and a background in health specialties. This finding aligns with various local studies conducted across different regions in Saudi Arabia. It was anticipated, as BLS-related knowledge is typically integrated into the curricula of students in health specialty programs and is often a graduation requirement [6,23]. Additionally, another study revealed that knowledge of BLS was highest not only among health-related students but also among those in colleges where BLS courses were a fundamental component of the curriculum. A study concluded that incorporating BLS information into university curricula enhances awareness of BLS, regardless of the nature of the college [24]. Individuals with higher economic income levels demonstrated a notably superior awareness of BLS in this study. This finding underscores the influence of socioeconomic factors on healthcare knowledge and preparedness. It suggests that individuals with greater economic resources may have more access to educational opportunities or resources related to BLS training. This correlation highlights the importance of targeted educational initiatives and accessibility to BLS courses for a wider demographic, particularly those from lower economic backgrounds, to ensure that lifesaving skills are accessible to all members of the community, regardless of financial status [25,26].The attitude towards enrolling in the BLS course was notably positive in our study, with 88% of participants expressing interest in undertaking the course. This finding resonates with previous research. ALSharari et al. discovered that 90% of their study's participants expressed a desire to learn BLS, while Alanazi et al. reported similar results, with 67% of participants expressing a willingness to learn BLS. Moreover, a study conducted by Alazmi and Alzahrani in 2020, which focused on secondary school teachers, found that 78.4% were inclined to take a BLS course. These consistent findings reflect a generally positive attitude toward acquiring BLS skills among Saudis, regardless of the specific demographic or educational background of the individuals involved [26,27]. Females and students in health-related fields exhibited a significantly higher inclination to enroll in BLS courses, as evidenced by the findings of this study. This aligns with the notion that individuals with a background in health-related disciplines may inherently recognize the value and necessity of BLS training due to their exposure to healthcare concepts and agree with other local studies [6]. Additionally, the higher willingness among female participants could be attributed to a heightened sense of responsibility toward the well-being of others, a characteristic often associated with females. These results emphasize the importance of targeted educational initiatives aimed at enhancing BLS awareness, particularly among males and those in non-health-related fields, to ensure a more widespread and well-prepared community in emergencies.Participants in the study identified several barriers and incentives affecting their decision to enroll in a BLS course. Among the barriers, concerns over the time commitment required for the course were raised. Additionally, a lack of familiarity with the locations where the course is offered was mentioned as a potential obstacle. Some participants expressed hesitance due to their perceived age-related limitations, while financial considerations were also cited, indicating that the cost of the course could be a deterrent. Furthermore, a notable percentage of participants felt that the absence of a compulsory requirement to take the course could influence their decision. Some individuals expressed concerns about the difficulty level of the course, suggesting that perceived complexity might deter participation [16,25,26].On the other hand, participants also acknowledged several incentives for enrolling in a BLS course. Many expressed a personal interest in acquiring these life-saving skills, highlighting a genuine desire to be able to respond effectively in emergencies. For those in health-related fields, the course's relevance to their specialty was a compelling motivator. Additionally, a significant number of participants recognized the course as a mandatory component for their chosen careers, underlining its critical importance [23]. Another key incentive was the belief that obtaining BLS certification would equip them with the ability to assist others in critical situations. Furthermore, some participants had personal experiences in situations where BLS skills were needed, reinforcing the value and necessity of such training [16,26].

The identified barriers and incentives provide valuable insights into the factors influencing individuals' decisions regarding enrolling in a BLS course. The mentioned barriers reflect common concerns that potential participants may have. Addressing these issues is crucial in making BLS courses more accessible and appealing. For example, providing flexible scheduling options, ensuring clear and widely available information about course locations, and offering financial assistance or affordable options can help alleviate some of these concerns. Conversely, the incentives highlighted demonstrate the various motivations that drive individuals to pursue BLS training. Personal interest, career relevance, and the desire to be prepared for emergencies are strong motivating factors. Recognizing and leveraging these incentives in course promotion and curriculum design can enhance participation rates and ultimately contribute to a more prepared and responsive community in emergencies. Overall, understanding these barriers and incentives is instrumental in tailoring BLS programs to meet the specific needs and motivations of potential participants, ultimately leading to a more informed and capable community in handling critical situations [23,28-30]. This study has some limitations, whilst it estimated the level of awareness it did not assess practical BLS skills among students. Furthermore, most of the respondents were female and the sample size was limited to only Riyadh universities. Another limitation is the use of the snowball sampling method, which may result in selection bias and compromise the representativeness of the sample, potentially limiting the generalizability of the results.

## Conclusions

The present research indicates that while Riyadh Universities’ students exhibit a positive attitude towards learning BLS, there is a noticeable lack of BLS knowledge, particularly among male and non-health specialties’ students. These findings highlight the importance of establishing academic BLS training programs as a graduation requirement for high school and university students across different specialties, not limited to health-related fields. BLS for Lay Rescuers: This curriculum is tailored for individuals who are a non-healthcare professional but may need to respond to a cardiac emergency in their workplaces or communities. It covers recognizing cardiac arrest, basic CPR skills, the use of an AED, and pediatric BLS.

## References

[1] Bogle BM, Ning H, Mehrotra S, Goldberger JJ, Lloyd-Jones DM (2016). Lifetime risk for sudden cardiac death in the community. J Am Heart Assoc.

[2] Narayan DP, Biradar SV, Reddy MT, Bk S (2015). Assessment of knowledge and attitude about basic life support among dental interns and postgraduate students in Bangalore city, India. World J Emerg Med.

[3] Albazee E, Alnifise M, Almahmoud L (2022). Basic life support awareness level among medical students in Jordan: a cross-sectional study. Front Emerg Med.

[4] Bin Salleeh HM, Gabralla KA, Leggio WJ, Al Aseri ZA (2015). Out-of-hospital adult cardiac arrests in a university hospital in central Saudi Arabia. Saudi Med J.

[5] Buck-Barrett I, Squire I (2004). The use of basic life support skills by hospital staff; what skills should be taught?. Resuscitation.

[6] Alsharari AO, Alduraywish A, Al-Zarea EA, Salmon NI, Sheikh MSA (2018). Current status of knowledge about cardiopulmonary resuscitation among the university students in the northern region of Saudi Arabia. Cardiol Res Pract.

[7] Yan S, Gan Y, Jiang N (2020). The global survival rate among adult out-of-hospital cardiac arrest patients who received cardiopulmonary resuscitation: a systematic review and meta-analysis. Crit Care.

[8] (2005). Introduction. Circulation.

[9] Al Harbi N, Afifi A, Alateeq M, Tourkmani A, Alharbi T, Albattal S (2018). Awareness of basic life support and cardiopulmonary resuscitation among female secondary school students in government schools in Riyadh city, KSA. J Family Med Prim Care.

[10] Al Enizi BA, Saquib N, Zaghloul MS, Alaboud MS, Shahid MS, Saquib J (2016). Knowledge and attitudes about basic life support among secondary school teachers in Al-Qassim, Saudi Arabia. Int J Health Sci (Qassim).

[11] Hasselqvist-Ax I, Riva G, Herlitz J (2015). Early cardiopulmonary resuscitation in out-of-hospital cardiac arrest. N Engl J Med.

[12] Plant N, Taylor K (2013). How best to teach CPR to schoolchildren: a systematic review. Resuscitation.

[13] Jarrah S, Judeh M, AbuRuz ME (2018). Evaluation of public awareness, knowledge and attitudes towards basic life support: a cross-sectional study. BMC Emerg Med.

[14] Subki AH, Mortada HH, Alsallum MS (2018). Basic life support knowledge among a nonmedical population in Jeddah, Saudi Arabia: cross-sectional study. Interact J Med Res.

[15] Roy Chowdhury S, Anantharaman V (2021). Public attitudes towards cardiopulmonary resuscitation training and performance in Singapore. Int J Emerg Med.

[16] Khashaba A, Alharbi M, Alghunaim M (2021). Knowledge and awareness of basic life support among nonhealth-care providers in Riyadh. Saudi J Oral Sci.

[17] Al-Mohaissen MA (2017). Knowledge and attitudes towards basic life support among health students at a Saudi women’s university. Sultan Qaboos Univ Med J.

[18] Jamalpour MR, Asadi HK, Zarei K (2015). Basic life support knowledge and skills of Iranian general dental practitioners to perform cardiopulmonary resuscitation. Niger Med J.

[19] Ghanem E, Elgazar M, Oweda K (2018). Awareness of basic life support among Egyptian medical students; a cross-sectional study. Emergency.

[20] Chandrasekaran S, Kumar S, Bhat SA, Saravanakumar Saravanakumar, Shabbir PM, Chandrasekaran V (2010). Awareness of basic life support among medical, dental, nursing students and doctors. Indian J Anaesth.

[21] Phillips PS, Nolan JP (2001). Training in basic and advanced life support in UK medical schools: questionnaire survey. BMJ.

[22] Vausedvan B, Lucas A, M G, Bhaskar A, Areekal B (2016). Assessment of level of knowledge of basic life support algorithm among medical and nursing students in a tertiary care teaching hospital. Int J Community Med Public Health.

[23] Awadalla NJ, Al Humayed RS, Mahfouz AA (2020). Experience of basic life support among king khalid university health profession students, southwestern Saudi Arabia. Int J Environ Res Public Health.

[24] Ahmad A, Akhter N, Mandal RK (2018). Knowledge of basic life support among the students of Jazan University, Saudi Arabia: is it adequate to save a life?. Alexandria J Med.

[25] Alazmi AA, Alzahrani FA (2020). Knowledge and attitude of basic life support in the community of Dammam City, Saudi Arabia. Int J Sci Study.

[26] Alghamdi YA, Alghamdi TA, Alghamdi FS, Alghamdi AH (2021). Awareness and attitude about basic life support among medical school students in Jeddah University, 2019: a cross-sectional study. J Family Med Prim Care.

[27] Alanazi A, Bin-Hotan Bin-Hotan, ALqahtani M (2013). Community awareness about cardiopulmonary resuscitation among secondary school students in Riyadh. World J Med Sci.

[28] Saquib SA, Al-Harthi HM, Khoshhal AA (2019). Knowledge and attitude about basic life support and emergency medical services amongst healthcare interns in university hospitals: a cross-sectional study. Emerg Med Int.

[29] Almojarthe B, Alqahtani S, AlGouzi B, Alluhayb W, Asiri N (2021). Awareness of secondary school students regarding basic life support in Abha City, Southern Saudi Arabia: a cross-sectional survey. Sci World J.

[30] Willmore RD, Veljanoski D, Ozdes F (2019). Do medical students studying in the United Kingdom have an adequate factual knowledge of basic life support?. World J Emerg Med.

